# A new risk-stratification system for hepatoblastoma in children under six years old and the significance for prognosis evaluation—a 14-year retrospective study from a single center

**DOI:** 10.1186/s12885-021-08095-x

**Published:** 2021-04-13

**Authors:** Tian Zhi, Wei-Ling Zhang, Yi Zhang, Hui-Min Hu, Yi-Zhuo Wang, Dong-Sheng Huang

**Affiliations:** Department of Pediatrics, Beijing Tongren Hospital Capital Medical University, No. 1 Dongjiaominxiang, Dongcheng District, Beijing, 100730 China

**Keywords:** Hepatoblastoma, Children, Risk factor, AFP, PRETEXT staging system, Prognosis, Chemotherapy

## Abstract

**Background:**

This study explores and analyzes the clinical characteristics and prognostic factors of hepatoblastoma (HB) in children under 6 years old and establishes a new risk-stratification system for individualized therapy.

**Methods:**

The clinical data of 382 pediatric patients under 6 years old (231 males and 151 females) who had been diagnosed with HB by pathology between May 2005 and May 2019 were collected. By analyzing the risk factors influencing the survival rate of patients with HB, a new risk-stratification system was established, and it was compared with previous risk-stratification systems by a receiver operating characteristic (ROC) curve.

**Results:**

1. According to a Kaplan-Meier survival analysis, the one-year, three-year, and five-year overall survival (OS) was 93.7, 84.0, and 73.9%, respectively, and the event-free survival (EFS) was 90.5, 79.2, and 67.5%, respectively.
2. The independent risk factors influencing prognosis in pediatric patients with HB were alpha-fetoprotein (AFP) < 100 ng/ml or > 1000 ng/ml (HR = 3.341, *P* = 0.005); platelet count > 400 × 10^9^/L (pooled hazard ratio [HR] = 2.123, *P* = 0.026); PRETEXT stage IV (HR = 4.026, *P* = 0.001); vascular involvement (HR = 2.178, *P* = 0.019); distant metastasis (HR = 2.634, *P* = 0.010);and multifocality (HR = 2.215, *P* = 0.012).
3. A new risk-stratification system was established and divided into three groups: low risk, moderate risk, and high risk. There were statistical differences among the three groups (*P* = 0.002). Compared with the previous risk-staging systems, there was no significant difference in the survival rate. Although the effect in the guiding therapy was the same, the area under the curve for the ROC curve was 0.835 (95% CI: 0.784–0.885) for the new stratification system.

**Conclusion:**

This new risk-stratification system had a better predictive value for the prognosis of pediatric patients with HB than other stratification systems.

**Supplementary Information:**

The online version contains supplementary material available at 10.1186/s12885-021-08095-x.

## Background

Hepatoblastoma (HB) is the most common hepatic cancer in children, accounting for 50 to 60% of cases [1, 2]. It is more common in infants, especially in children under 3 years old [3], and affects the patient’s quality of life and prognosis for survival. In recent years, with the deepening understanding of the biological characteristics of tumors and the improvement of neoadjuvant chemotherapy, the disease-free survival rate in low-risk pediatric patients has reached 80 to 90% [4]. But for high-risk patients, especially those with distant metastasis, the treatment is difficult, and the prognosis is poor. Thus, an effective risk-stratification system is important for guiding the treatment and predicting the prognosis.

We studied 382 HB patients under 6 years old who had been diagnosed in our center between May 2005 and May 2019. The clinical data were analyzed retrospectively to examine the risk factors affecting the prognosis. A new risk-stratification system was established to provide better guidance for prognosis evaluation and clinically individualized therapy.

## Methods

### Patients

A total of 382 children with HB admitted to Department of Beijing Tongren Hospital affiliated to Capital Medical University from May 2005 to May 2019 were collected.

Inclusive criteria: ① all cases were diagnosed as hepatoblastoma by pathological examination of primary liver tumor; ② the age ranged from 0 to 6 years; ③ all cases were followed up completely.

Exclusion criteria: ① patients with severe liver and kidney dysfunction, cardiac dysfunction or drug intolerance; ② patients with other tumors or other underlying diseases; ③ patients were lost to follow-up.

All relevant examinations and treatments were approved by their guardians and signed informed consent, and were approved by Beijing Tongren Hospital affiliated to Capital Medical University ethics committee approval.

### Risk stratification

According to the international risk classification standards of HB, and based on the PRE-Treatment EXTent of tumor (PRETEXT) staging [5] (Table 1), the pediatric patients with HB were divided into the standard-risk group and the high-risk group in the combination of the serum AFP level and tumor invasion by the SIOPEL group [6]. The COG collaborative group divides pediatric patients into four groups: extremely low risk, low risk, moderate risk, and high risk [7]. The details are illustrated in Table 2.

**Table 1:** The PRETEXT preoperative staging system

Stage	Definition
Stage I	3 contiguous hepatic sections are free of tumor
Stage II	2 contiguous hepatic sections are free of tumor
Stage III	1 contiguous hepatic section is free of tumor
Stage IV	Tumor affects all four hepatic sections (almost always multifocal or infiltrative)

**Table 2:** The risk groups of HB defined by different collaboration groups

Risk grouping	Grouping criteria
**SIOPEL collaboration groups**	
standard-risk group	PRETEXT I、II or III stage, and AFP > 100 ng/ml
high-risk group	PRETEXT IV stage, orPRETEXT I、II or III stage with P(portal venous infiltration), V(Hepatic venous or vena cava invasion), E(Intrahepatic metastases), H(tumor rupture), M(distant location), N(Lymph node metastasis), and(or)AFP < 100 ng/ml
**COG collaboration groups**	
Extremely low risk group	Children of simple fetal type and children whose tumor can be surgically removed in PRETEXT I or II stage
Low risk group	Children of other pathology type and children whose tumor can be surgically removed in PRETEXT I or II stage
Moderate risk group	Children of other pathology type and who can not be surgically removed in PRETEXT II、III or IV stage
High risk group	Any child with metastatic lesions or AFP < 100 ng/ml

### Comprehensive therapeutic protocols

Pediatric patients with HB were treated with surgery and chemotherapy. For some patients with ruptured tumors, transarterial embolization or transcatheter arterial chemoembolization was necessary to control the intraperitoneal hemorrhage. After the hemorrhage was controlled, the corresponding risk-stratification treatment was carried out. The patients without a distant metastasis or patients with a very low risk or a few low risk underwent a first-stage surgical resection and postoperative chemotherapy, as appropriate. Some patients with low or moderate risk received two to four cycles of pre-operation chemotherapy, liver transplantation (LTx) or primary tumor resection by hepatectomy, and four to six cycles of consolidation chemotherapy after the operation.

Patients with a high risk and distant metastasis were treated with pre-operation chemotherapy. If the distant metastasis was reduced, a primary tumor resection, hepatectomy or LTx was performed, followed by postoperative chemotherapy. The routine first-line chemotherapy regimens were cisplatin + fluorouracil + vincristine (the C5V protocol); cisplatin + adriamycin (the PLADO protocol); or ifosfamide + carboplatin + pirarubicin + etoposide.

**Fig. 1:**
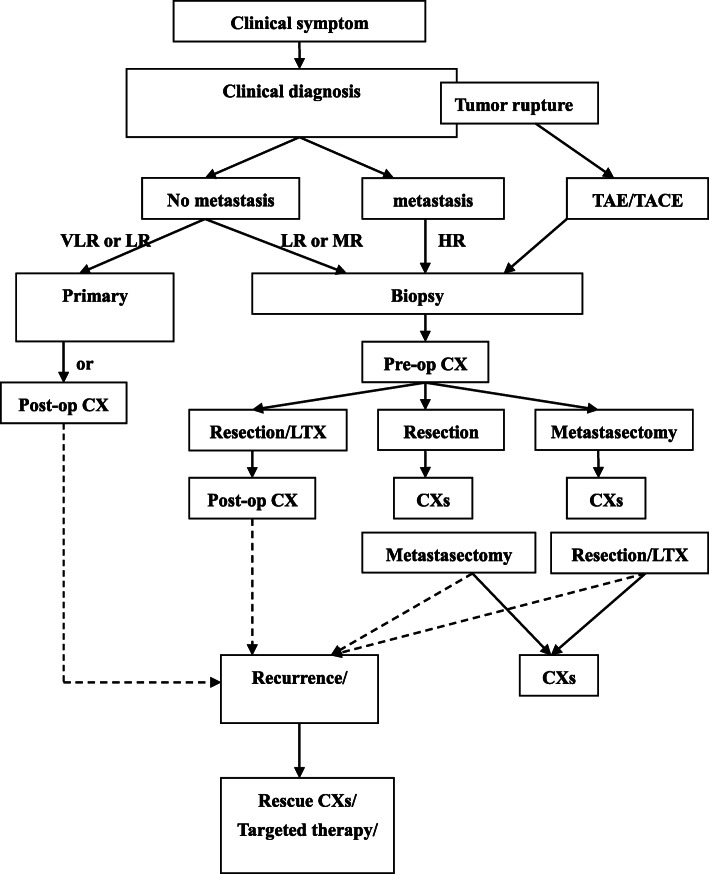
The comprehensive therapeutic protocols of HB

LTx might be considered for pediatric patients with multiple tumor foci in four hepatic regions, portal vein invasion, minor metastasis after neoadjuvant chemotherapy, a tumor that was unresectable by traditional surgery, residual tumor cells in the liver after surgery, or tumor recurrence after the operation. The cycle of chemotherapy might be prolonged in pediatric patients with recurrent tumor, advanced tumor, or refractory HB. Individualized chemotherapy might be performed: (1) irinotecan + cyclophosphamide + cisplatin + vincristine; (2) etoposide + cisplatin + pirarubin; or (3) cyclophosphamide + cisplatin + pirarubicin. Other therapies might be used (Fig. 1): high-dose chemotherapy (melphalan + cyclophosphamide + etoposide) combined with autologous peripheral blood stem-cell transplantation, targeted therapy, or molecular biological therapy. Mesna (2-mercaptoethanesulfonic acid sodium salt) was used as a rescue after the administration of cyclophosphamide.

### Monitoring indicators, follow-up, and evaluation criteria

Serum AFP was measured before each chemotherapy, and a peripheral blood routine test was performed during the procedure. The images (ultrasonography and computerized tomography) of the primary foci and/or the metastatic lesions were monitored every two cycles of chemotherapy. Follow-ups took place by telephone and by return visits to the hospital for re-examination. According to the follow-up results, the overall survival (OS:Time from randomization to death) and event-free survival (EFS: Time from randomization to relapse, progression, or death) were calculated, and the potential risk factors affecting the prognosis were analyzed.

### Statistical methods

SPSS 20.0 software was used for data analysis. The counting data are expressed as percentage (%). The measurement data were expressed as mean ± standard deviation. A χ^2^ test was used for comparison between groups, and the Kaplan-Meier method was used for survival analysis. The log-rank test was adopted for the comparison of survival rates among subgroups. The factors statistically significant at a *p* value below 0.01 in the univariate analysis were chosen for multivariable analysis in a Cox risk regression model As a result of the study, a new prognostic stratification system was established. The area under the ROC curve (AUC) was compared with the previous risk classification systems, and the predictive function was evaluated. *P* < 0.05 was considered statistically significant.

## Results

### Patients and tumor characteristics

A total of 382 cases were included in this study, including 231 males and 151 females, the clinical features were shown in Table 3. The ages ranged between 0.08 and 5.92 years, with a median age of 1.75 years. In the study group, 128 patients (33.5%) were younger than 1 year; 184 patients (48.2%) were one to 3 years old; and 70 patients (18.3%) were between three and 6 years old. The most common initial symptom was an abdominal mass, occurring in 266 patients (69.6%). Fifty-one patients (13.3%) had an initial symptom of abdominal pain and distention. The other manifestations were vomiting and poor appetite in 32 patients (8.4%), fever and cough in 18 patients (4.7%), jaundice in nine patients (2.4%), and emaciation and anemia in six patients (1.6%).

**Table 3:** The clinical characteristics and univariate analysis of 382 HB children^a^

Group		n	Percent(%)	χ^**2**^	***P*** value
Sex	Male	231	60.5	2.766	0.633
	Female	151	39.5		
Age (years)	<1	128	33.5	9.053	0.021
	1–3	184	48.2		
	> 3 and < 6 y	70	18.3		
AFP at first visit (ng/ml)	<100	25	6.5	12.697	<0.001
	100–1000	120	31.4		
	>1000	237	62.1		
Platelet count at first visit (×10^9^/L)	≤400	167	43.7	11.367	0.001
	>400	215	56.3		
Pathological classification	Epithelial type	213	55.8	8.535	0.013
	Mixed	169	44.2		
PRETEXT staging	I period	9	2.4	19.233	<0.001
	II period	97	25.4		
	III period	224	58.6		
	IV period	52	13.6		
Portal venous involvement (P) and/or hepatic venous/IVC^b^ involvement (V)	Yes	86	22.5	14.643	<0.001
	No	296	77.5		
Extrahepatic tumor extension (E)	Yes	73	19.1	4.872	0.459
	No	309	80.9		
Tumor rupture (R)	Yes	24	6.3	2.176	0.251
	No	358	93.7		
Metastasis (M)	Yes	171	44.8	9.766	0.001
	No	211	55.2		
Multifocality (F)	Yes	96	25.1	9.041	0.002
	No	286	74.9		
Complete resection of primary tumor	Yes	283	74.1	1.690	0.345
	No	99	25.9		

^a^It was a univariate Cox regression of five-year OS separately on each of the clinical characteristic

^b^*IVC* inferior vena cava

The mean value of alpha-fetoprotein (AFP) at the first visit was 97,406.5 ± 11,214.8 ng/ml, with the maximum value 484,000 ng/ml and the minimum value 25.8 ng/ml, which were all higher than the normal range (0–20 ng/ml). Most of the patients had an AFP > 1000 ng/ml (237 patients, 62.1%). The mean value of the platelet count (PLT) was (408 ± 224) × 10^9^/L, with a maximum value of 1550 × 10^9^/L and a minimum value of 65 × 10^9^/L. The majority of the patients had a PLT > 400 × 10^9^/L (215 cases, 56.3%).

According to the tumor tissue morphology defined by the Children’s Hepatoma International Collaboration (CHIC) [8, 9], epithelial tissue was the primary pathological tissue type in the present study, accounting for 55.8% (213/382) cases. Within the epithelial-type cases, 78 cases were the fetal type (36.6%), 119 were embryonic (55.9%), 11 were giant-beam (5.2%), and five were undifferentiated small-cell (2.3%). There were 169 mixed-type cases (44.2%).

According to the PRETEXT staging system proposed by the International Childhood Liver Tumors Strategy Group (SIOPEL) [10], 224 patients (58.6%) were stage III, and 52 patients (13.6%) were stage IV. At the initial visit, 86 patients (22.5%) had involvement of the portal vein, hepatic vein, or vena cava; 73 patients (19.1%) had extrahepatic tumor extension; 24 patients (6.3%) had tumor rupture; and 171 patients had a distant metastasis. The most common site of metastasis was the lung 139/171 (81.3%), and metastasis sites were also found in the brain, bone, bone marrow, and spinal canal. Multifocality was found in 96 patients (25.1%).

### Follow-ups and survival results

**Fig. 2:**
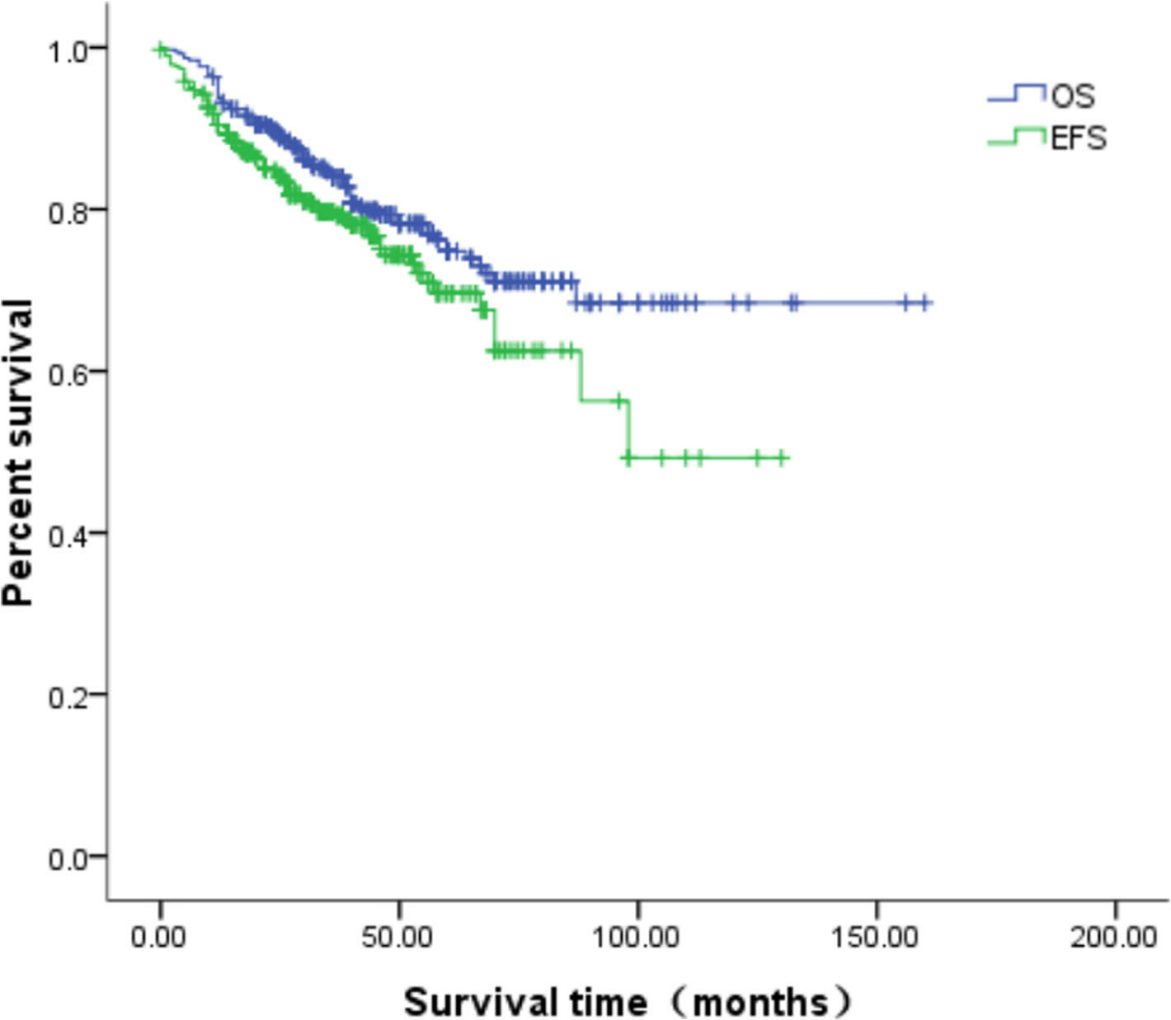
The curves of the OS and EFS of 382 pediatric patients with HB

Follow-ups continued until May 2020, with a total follow-up duration of 1 to 167 months (median 56 months). Among the study patients, 218 patients achieved complete remission, and 69 patients had partial remission, for an effective rate (effective rate = (complete remission cases + partial remission cases) / total cases) of 75.1% (287/382). HB progressed in 16 patients, and 79 patients died. According to the Kaplan-Meier survival analysis, the one-year, three-year, and five-year OS was 93.7, 84.0, and 73.9%, respectively. The EFS was 90.5, 79.2, and 67.5%, respectively (Fig. 2).

### Analysis of prognostic risk factors

**Fig. 3:**
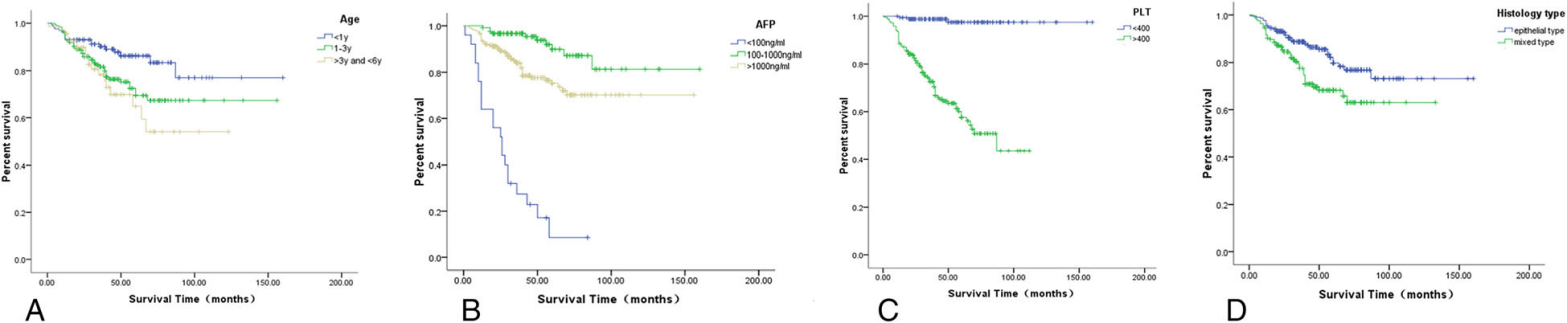
The correlation between different risk factors and the prognosis. **a** The effects of different age of onset on the prognosis. **b** The effects of the AFP levels at the initial diagnosis on the prognosis. **c** The effects of the PLT count (×10^9^/L) at the initial diagnosis on the prognosis. **d** Comparison of OS of patients with different pathological types

The results of the log-rank test showed that patients under the age of 1 year had a better five-year OS (89.0%) than patients over the age of 3 years (54.1%) and the difference was statistically significant (*P* = 0.021). This indicates that an increased age of diagnosis correlates with a lower survival rate (Fig. 3, a). AFP and PLT levels were measured at the time of diagnosis. Patients in the AFP < 100 ng/ml group had the lowest OS (17.1%), while the prognosis of HB patients with the AFP between 100 and 1000 ng/ml was the best (five-year OS 93.7%). Patients with a PLT > 400 × 10^9^/L had a lower OS (60.1%) than those with a PLT of ≤400 × 10^9^/L (OS 93.5%, *P* < 0.01, Figs. 3, b and c).

For patients whose primary pathological tissue type was in the epithelial tissue, the OS was higher (82.2%) than that for patients who had a mixed type (68.2%) (*P* = 0.013, Fig. 3, d). The prognosis for patients with an undifferentiated small-cell type was relatively worse than the prognosis for other epithelial types.

**Fig. 4:**
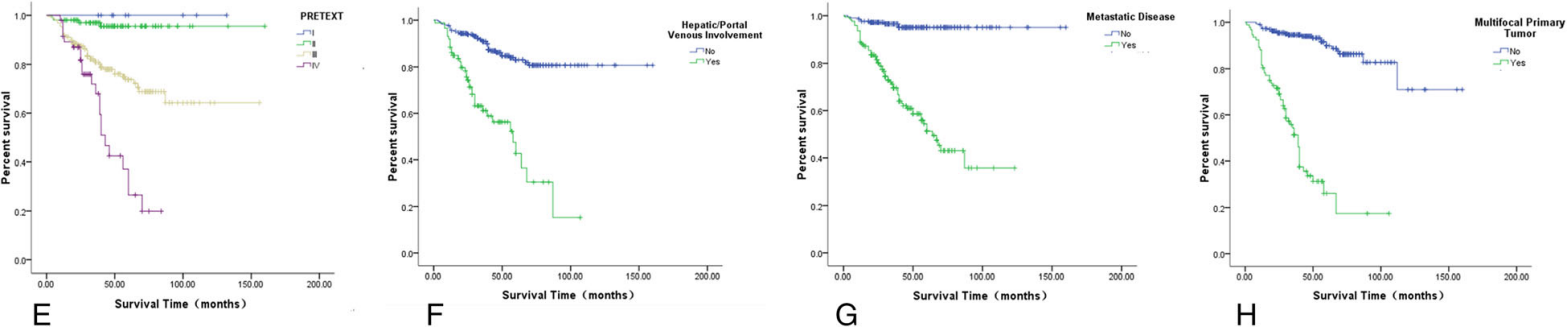
The correlation between different risk factors and the prognosis. **e** Comparison of OS of patients with different PRETEXT stages. **f** Comparison of OS of patients with or without vascular invasion. **g** Comparison of OS of patients with or without distant metastasis. **h** Comparison of OS of patients with a single focus or multiple foci of the primary tumor

The PRETEXT staging system also indicated statistical significance: the five-year OS of patients in the PRETEXT stage IV group (high-risk) was only 26.5%, significantly lower than those in the PRETEXT stages I, II, or III groups (*P* < 0.01, Fig. 4,e-h). In addition, the five-year OS of patients with vascular involvement (portal vein, hepatic vein, or vena cava), distant metastasis, or multifocality were 47.5, 51.3, and 26.1%, respectively, and the prognoses were all poor (*P* < 0.05, Figs. 4, e-h). However, in the present study, there was no significant correlation between the survival rate and sex, presence of tumor rupture, extrahepatic tumor extension or complete resection of the primary tumor which means there is no residue in liver under microscope. (*P* > 0.05) (Table 3).

The clinical factors described above were introduced into the Cox regression model for multivariate analysis. The results showed the most significant independent risk factors for the prognosis of pediatric patients with HB were AFP levels, platelet counts, PRETEXT stage IV, presence of vascular involvement, distant metastasis, and multifocality (Table 4 and the supplemental figures).

**Table 4:** The multivariate risk analysis of the COX regression model

Risk factors	n	HR	95%IC	***P*** values
AFP < 100 ng/ml or > 1000 ng/ml	262	3.341	1.742–4.475	0.005
PLT > 400 × 10^9^/L	215	2.123	0.917–3.053	0.026
PRETEXT stage IV	52	4.026	2.063–4.442	0.001
Vascular involvement(V/P)	86	2.178	1.584–4.012	0.019
Metastasis	171	2.634	1.689–3.932	0.010
Multifocality	96	2.215	1.487–4.196	0.012

### Establishment of a new risk-stratification system

**Fig. 5:**
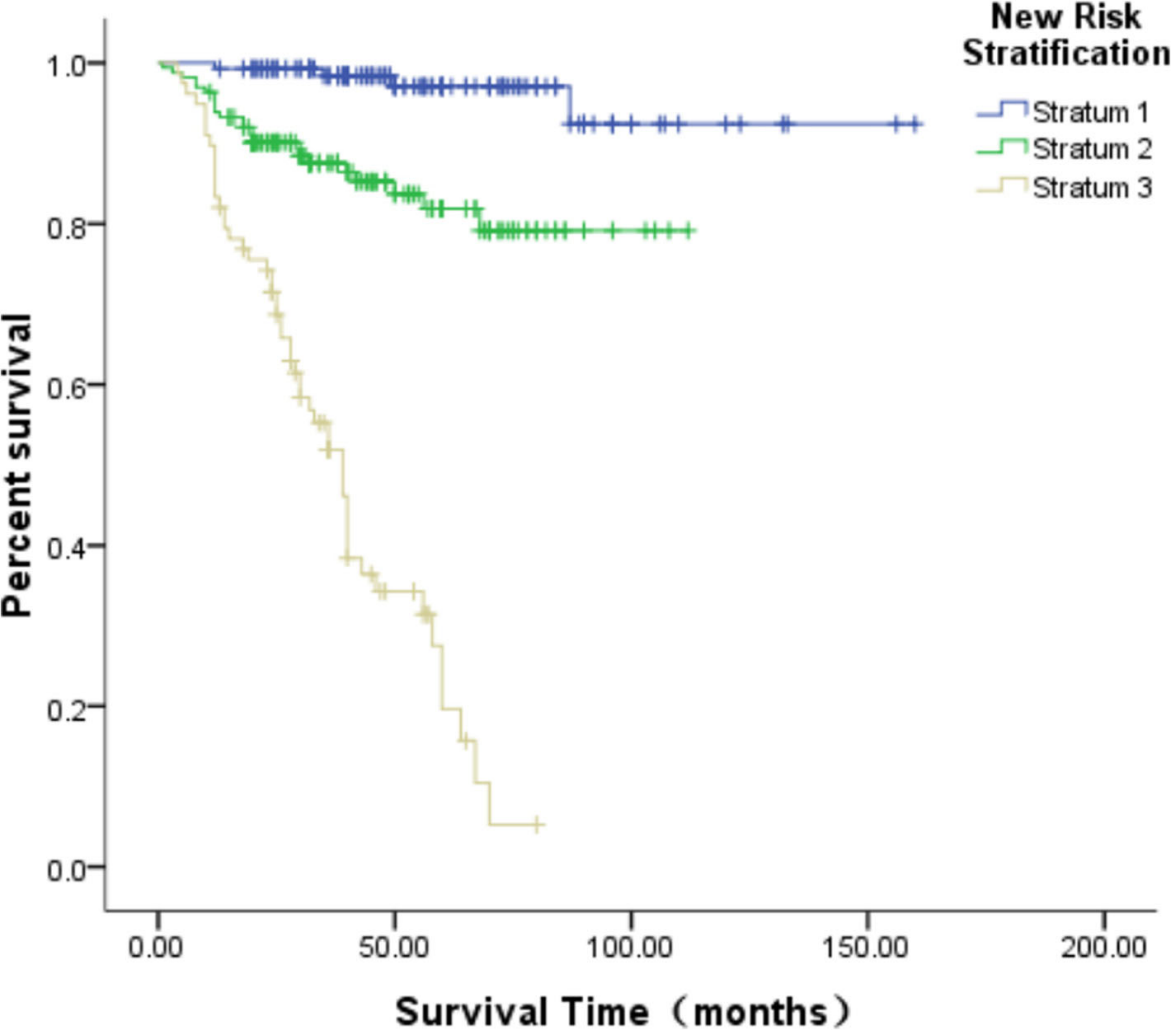
Comparison of the OS between different groups according to the new HB risk stratification system

To better carry out the clinical management of HB, the present study established a new risk-stratification system. For each of the 6 factors which were statistically significant in the multivariable Cox regression, a patient was assigned a score of 0 (absent) or 1 (present). The total risk score of each patient was then calculated as the sum of the 6 scores. Fifty-eight patients (15.2%) had a score of zero points, 80 cases (20.9%) a score of one point, 96 cases (25.1%) a score of two points, 67 cases (17.5%) a score of three points, 44 cases (11.5%) a score of four points, 26 cases (6.8%) a score of five points, and 11 cases (3.0%) a score of six points. The scores were then divided into three groups: the low-risk group has a score of zero to one point, the moderate risk group two to three points, and the high-risk group four to six points. Using this new risk-stratification system to analyze the 382 patients in the present study, 138 patients were in the low-risk group, 163 patients were in the moderate-risk group, and 81 patients were in the high-risk group. The five-year OS of each group is shown in Fig. 5, and there were significant statistical differences among the three groups (*P* = 0.002).

**Fig. 6:**
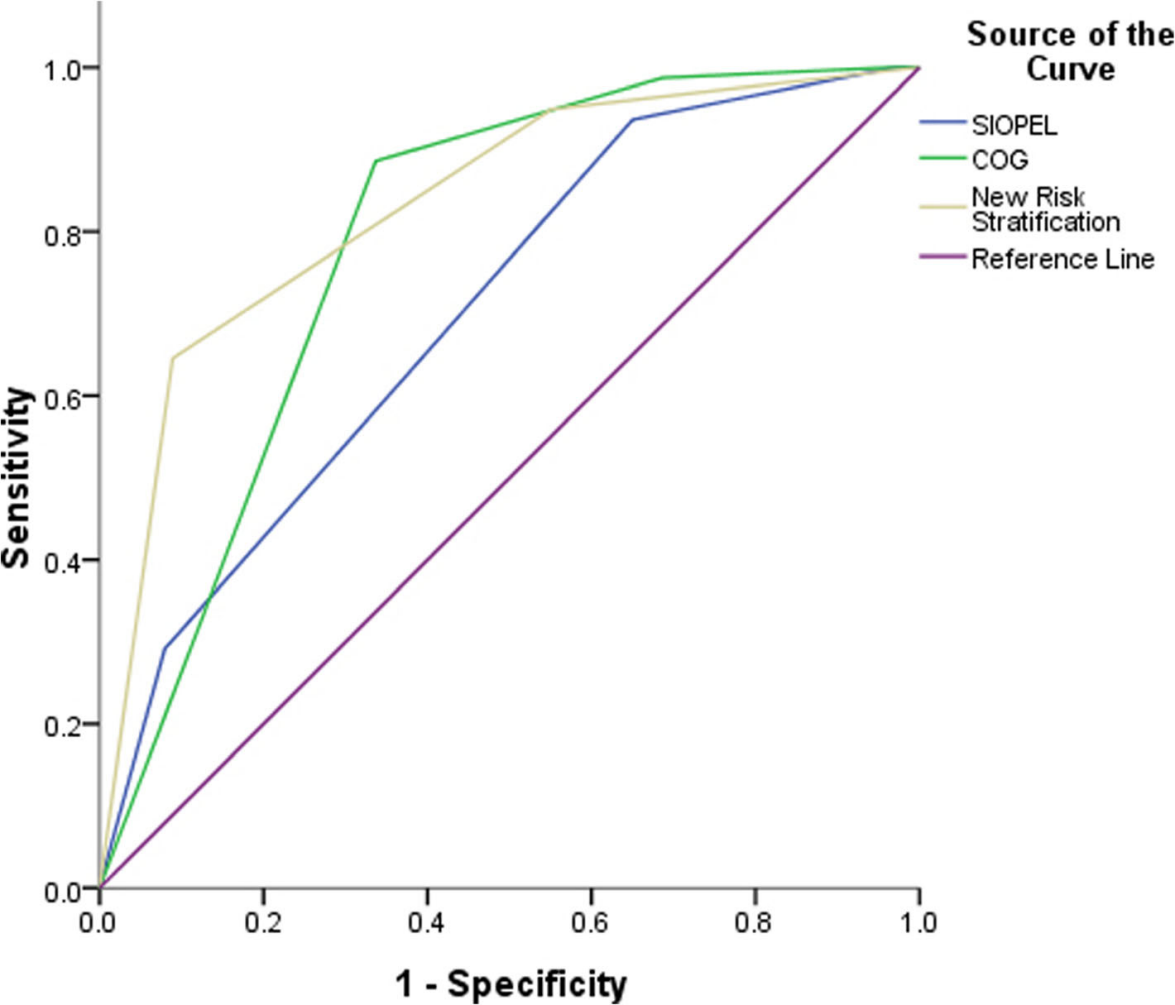
Comparison of the survival prediction value between the new HB risk stratification system and the previous classification systems

The clinical distribution of the newly established risk-stratification system in the present study was compared with the risk-grouping standards of the SIOPEL and COG collaborative groups, and the distribution proportion and survival rate are shown in Table 5.This demonstrates that the three risk-stratification systems show relatively similar survival rates after treatment among the different groups (*P* > 0.05), which means that there are no significant differences among the three risk-stratification systems. However, the ROC curve (Fig. 6) revealed that the AUC of the new risk-stratification system is 0.835 (95% CI: 0.784–0.885), which is significantly higher than that of the risk groups defined by SIOPEL (AUC: 0.702, 95% CI: 0.641–0.763, *P* = 0.023) and COG (AUC: 0.789, 95% CI: 0.741–0.836, *P* = 0.038), and the difference was statistically significant. This suggests that the new risk-stratification system may be better than the previous two systems in predicting the survival rate of pediatric patients with HB.

**Table 5:** Comparison of the new risk stratification system and the previous classification systems

Risk stratification system	n	Ratio(%)	N of death	Survival rate(%)
**SIOPEL collaboration groups**				
Standard-risk group	261	68.3	28	90.4
High-risk group	121	31.7	51	57.9
**COG collaboration groups**				
Extremely low risk group	10	2.6	0	100
Low risk group	86	22.5	3	96.5
Moderate risk group	114	29.9	17	85.1
High risk group	172	45.0	59	65.7
**New risk stratification system**				
Low risk group	138	36.1	5	96.4
Moderate risk group	163	42.7	25	84.7
High risk group	81	21.2	49	39.5

## Discussion

During the past 30 years, great progress has been made in the treatment of HB through multi-center collaborative research carried out by the four international groups: SIOPEL (Europe), COG (United States), JPLT (Japanese Study Group for Pediatric Liver Tumors), and the German Liver Tumor Study [11–14]. Brown et al. analyzed the prognosis of 154 pediatric HB patients from 30 countries and found that after comprehensive treatments, the five-year OS and EFS of HB were 75 and 66%, respectively [15]. The median follow-up duration in the present study was 56 months, the effective rate of treatment was 75.1%, the five-year OS and EFS were 73.9 and 67.5%, respectively, consistent with the data reported internationally.

The incidence of HB is still relatively high. Most pediatric patients have distant metastasis at the time of initial diagnosis, and there are more risk factors affecting the prognosis of HB. With the development of medical treatment and the improving understanding of the disease, the stratification of risk factors is still changing [16]. Huang et al. found that age has an important impact on prognosis [17]. and Haeberle et al. also believed that age plays an important role in the risk stratification of HB [18]. Based on the clinical data and risk factors of 382 cases in our center during the past 14 years, the author of the present study found that patients who were older at the time of diagnosis had a poorer prognosis for OS, consistent with the results of Maibach [9]. However, through multivariate analysis, it was shown that age was not an independent risk factor for the prognosis of HB in this study, perhaps because the pediatric patients enrolled had a narrow age range (less than 6 years old). Thus, the role of age should be further examined by expanding the age range in a future study.

As a tumor marker of HB, serum AFP plays an important role in patient prognosis [19]. In the present study, the prognosis of pediatric patients with AFP < 100 ng/ml or AFP > 1000 ng/ml was worse than that of pediatric patients with AFP in the range of 100 ng/ml to 1000 ng/ml (*P* < 0.05). This indicates that AFP is an independent risk factor for the prognosis of HB, which is consistent with the results of Von Schweinitz et al. [20]

The present study also confirmed that the increase of platelet counts was an independent risk factor affecting the prognosis of HB. In 2015, a study showed that the increase of platelet counts in the peripheral blood correlated with infection, inflammatory disease, and malignant tumors [21]. In clinical practice, the author of the present study also found that some cases with HB, especially those in stage IV or with distant metastasis, were more prone to have an abnormal increase in platelet count at the initial diagnosis.

The SIOPEL believes that the PRETEXT staging of pediatric patients with HB has an important prognostic value [9], especially for patients who do not undergo surgery, which can predict the resectability of the tumor, while the complete resection of the hepatic tumor is the key to the treatment of patients with HB. The present study confirms that PRETEXT stages I-IV significantly correlated with OS.

According to the results of the multivariate analysis of the Cox regression model, distant metastasis, hepatic vascular involvement, and multifocality were also independent risk factors for the prognosis of pediatric patients with HB.

The purpose of the present study was to establish a recognized and widely accepted risk-stratification scheme for the diagnosis of HB. This new system was based on the results of statistical tests and quantification of the above risk factors. Compared with the risk-stratification systems developed by the SIOPEL and COG collaborative groups, the new system was equally effective in guiding the treatment. The new risk-stratification system was superior to other risk-classification systems in predicting the prognosis via the ROC curve (AUC: 0.835).

### Limitation

However, the new risk-stratification system was based on retrospective research data and existing therapeutic patient protocols. This system now needs to be verified by a prospective study, which is currently being actively carried out.

According to Hiyama [22] and Wang [23], tumor rupture, incomplete resection of the tumor, and invasion of the adjacent tissues and organs outside the liver might also seriously affect the prognosis of HB. However, the results of the present study were not completely consistent with the literature. We speculate that because most of the patients with HB admitted in our center were in a late or refractory stage, this might influence the correlation of these risk factors to the survival rate. Moreover, there are data showing that factors such as maternal hypertension during pregnancy, excessive amniotic fluid, smoking history, and birth weight of < 1500 g can increase the incidence of HB [24]. These factors were not considered in the present study. A more scientific and complete risk-stratification system should be established in the future with more comprehensive clinical data. Any such retrospective analysis is bound to deliver a result which is over-optimistic with respect to other classification systems developed in different cohorts.

## Conclusion

The present study was preliminary. Our risk-stratification system should be studied with clinical data from a multi-center trial to establish the therapeutic protocols for each stratification. In the era of increased individual medication targeting, the treatment of HB can be individualized to improve the prognosis and survival rates of HB pediatric patients. This risk-stratification system can also provide a new template for the study of other rare tumors in both children and adults.

## Supplementary Information

**Additional file 1.**

## Data Availability

We declared that materials described in the manuscript, including all relevant raw data, will be freely available to any scientist wishing to use them for non-commercial purposes, without breaching participant confidentiality. The corresponding author Dong-Sheng Huang should be contacted if someone wants to request the data from this study.

## Abbreviations

HB: Hepatoblastoma
ROC: Receiver operating characteristic
OS: Overall survival
EFS: Event-free survival
AFP: Alpha-fetoprotein
PLT: Platelet count
CHIC: Children’s hepatoma international collaboration
PRETEXT: PRE-treatment EXTent of tumor
COG: Children’s oncology group
SIOPEL: The international childhood liver tumors strategy group
LTx: Liver transplantation
AUC: The area under the ROC curve
JPLT: Japanese study group for pediatric liver tumors
