# Supplementary material for: A new risk-stratification system for hepatoblastoma in children under six years old and the significance for prognosis evaluation—a 14-year retrospective study from a single center
Source: BMC Cancer. 2021 Apr 13;21:397. doi: 10.1186/s12885-021-08095-x (PMC8042704; doi:10.1186/s12885-021-08095-x)
Supplement: Supplementary file 1 — Additional file 1. [file 12885_2021_8095_MOESM1_ESM.docx]

Supplement figures

a
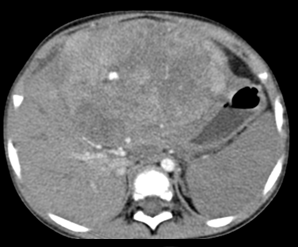

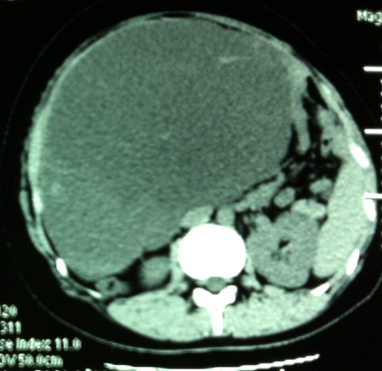
 b

c
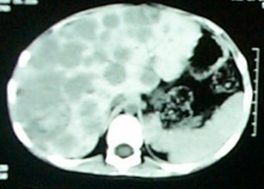

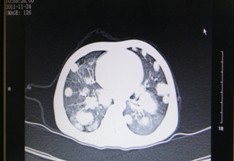
 d

e
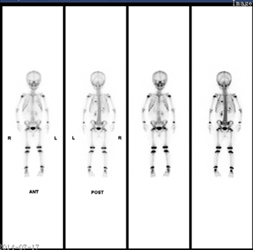

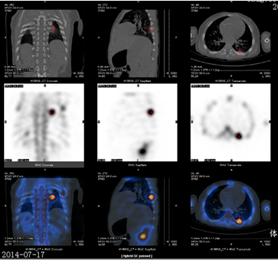
f

g
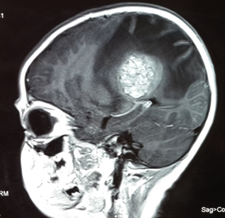

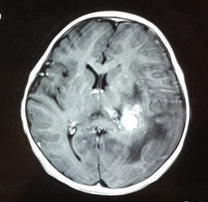
 h

i
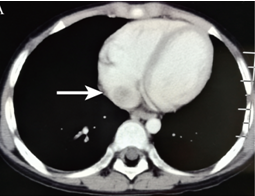

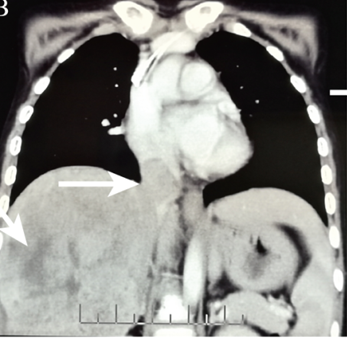
 j

These supplement figures illustrated the independent risk factors affecting the prognosis of pediatric patients with HB:

a-b PRETEXT stage IV: The abdominal CT in patients with HB showed a large occupying lesion of the liver, involving the four hepatic segments.

c Multiple intrahepatic foci: The abdominal CT showed multiple nodules of different sizes in the liver, indicating multiple foci of the tumor.

d-h Distant metastasis in different locations:

d The chest CT showed multiple metastatic nodules in both lungs.

e-f The whole body bone scan showed HB with bone metastasis, with a round radioactive distribution concentrated area in the 7th left rib and a horizontal flaky radioactive distribution increased area in the 11th right rib.

g-h The brain MR imaging indicated the intracranial metastasis.

i-j Vascular invasion: The chest CT showed that the tumor invaded the right atrium and the inferior vena cava.
